# Blood Reflux-Induced Epigenetic Factors HDACs and DNMTs Are Associated with the Development of Human Chronic Venous Disease

**DOI:** 10.3390/ijms232012536

**Published:** 2022-10-19

**Authors:** Shun-Fu Chang, Hsiao-En Tsai, Jong-Tar Kuo, Yu-Rong Ruan, Chiu-Yen Chen, Shin-Yi Wang, Po-Yu Liu, Ding-Yu Lee

**Affiliations:** 1 Department of Medical Research and Development, Chiayi Chang Gung Memorial Hospital, Chiayi 613, Taiwan; 2 Center for General Education, Chiayi Chang Gung University of Science and Technology, Chiayi 613, Taiwan; 3 Division of Cardiovascular Surgery, Department of Surgery, National Taiwan University Hsin-Chu Hospital, Hsinchu 300, Taiwan; 4 Department of Biological Science and Technology, China University of Science and Technology, Taipei 115, Taiwan; 5 Department of Bioscience and Biotechnology, National Taiwan Ocean University, Keelung 202, Taiwan; 6 Division of Infectious Diseases, Department of Internal Medicine, Taichung Veterans General Hospital, Taichung 407, Taiwan

**Keywords:** chronic venous disease, blood reflux, histone deacetylases, DNA methyltransferases

## Abstract

Blood reflux and metabolic regulation play important roles in chronic venous disease (CVD) development. Histone deacetylases (HDACs) and DNA methyltransferases (DNMTs) serve as repressors that inhibit metabolic signaling, which is induced by proatherogenic flow to promote aortic endothelial cell (EC) dysfunction and atherosclerosis. The aim of this study was to elucidate the relationship between blood reflux and epigenetic factors HDACs and DNMTs in CVD. Human varicose veins with different levels of blood reflux versus normal veins with normal venous flow were examined. The results show that HDAC-1, -2, -3, -5, and -7 are overexpressed in the endothelium of varicose veins with blood reflux. Blood reflux-induced HDACs are enhanced in the varicose veins with a longer duration time of blood reflux. In contrast, these HDACs are rarely expressed in the endothelium of the normal vein with normal venous flow. Similar results are obtained for DNMT1 and DNMT3a. Our findings suggest that the epigenetic factors, HDACs and DNMTs, are induced in venous ECs in response to blood reflux but are inhibited in response to normal venous flow. Blood reflux-induced HDACs and DNMTs could inhibit metabolic regulation and promote venous EC dysfunction, which is highly correlated with CVD pathogenesis.

## 1. Introduction

Chronic venous disease (CVD) is a common cardiovascular disease [1]. Blood reflux is a vital pathogenic factor that induces venous endothelial cell (EC) dysfunction and later progress to CVD [2,3]. Blood reflux is detected clinically to evaluate CVD, and a longer duration time of blood reflux is related to greater CVD severity [2,3]. Metabolic regulation, such as vitamin efficiency or supplementation, is also clinically associated with the development of CVD [4,5,6,7,8,9]. Vitamins can bind to their receptors to turn on signal transduction and regulate vascular functions [10,11,12]. This indicates that not only blood reflux but also metabolic regulation, plays a vital role in CVD.

Histone deacetylases (HDACs) and DNA methyltransferases (DNMTs) are major epigenetic factors that deacetylate and methylate, respectively, signaling molecules or transcription factors to regulate signal transduction [13,14]. Recent studies have shown that HDACs (Class I HDACs (HDAC-1, -2, and -3) and Class II HDACs (HDAC-5 and -7)) and DNMTs (DNMT1 and DNMT3a) serve as repressors that inhibit metabolic regulators (e.g., vitamin A receptor, vitamin D receptor, nitric oxide synthase (NOS), and NADPH quinone dehydrogenase 1 (NQO1)) to affect metabolic regulation in vascular cells [13,14,15,16,17,18,19,20,21,22,23,24]. In particular, HDACs and DNMTs are regulated by hemodynamic forces that affect metabolic regulation to promote pathogenesis in aortic endothelial cells (ECs) [13,14,19,20,21,22,23,24]. We reported that HDAC-1/-2/-3 and HDAC-3/-5/-7 can be induced by proatherogenic oscillatory flow to inhibit quinone metabolism and vitamin A signaling, respectively, to promote aortic endothelial dysfunctions [19,20]. Moreover, HDAC-3/-5/-7 are also modulated by proatherogenic oscillatory flow and antiatherogenic laminar flow, which affects the expression of KLF2, a vital transcription factor that regulates the metabolic state and nitric oxide (NO) production in aortic ECs [18,20,21]. In addition, DNMT1 and DNMT3a are induced by proatherogenic oscillatory flow to promote NO repression, inflammation, and proliferation, respectively, in aortic ECs [22,23,24]. In vivo animal models further link HDACs and DNMTs to the progression of aortic disease (atherosclerosis) [19,20,24]. Although HDACs and DNMTs are regulated by proatherogenic flow to modulate metabolic regulation and cell dysfunction in aortic ECs, whether they can be modulated by blood reflux to regulate venous EC dysfunction remains unknown. In addition, the roles of HDACs and DNMTs in CVD are not yet identified.

This study aimed to identify the in vivo expression of HDACs (Class I and II) and DNMTs in venous ECs in response to normal venous flow vs. blood reflux and to study their association with the development of CVD in humans. We collected normal human veins with normal venous flow and human varicose veins with different levels of blood reflux to mimic normal and pathogenic conditions, respectively, for comparison. These human specimens were used to identify the effects of normal venous flow vs. blood reflux in modulating the expression of HDACs and DNMTs. In addition, we define the in vivo roles of HDACs and DNMTs under normal and pathogenic conditions in human veins and correlate them with the development of human CVD.

## 2. Results

### 2.1. High Levels of Class I HDACs Are Expressed in Venous ECs in Response to Blood Reflux in Varicose Veins, but Low Levels Are Expressed in Response to Normal Venous Flow in Normal Veins

**Figure 1:**
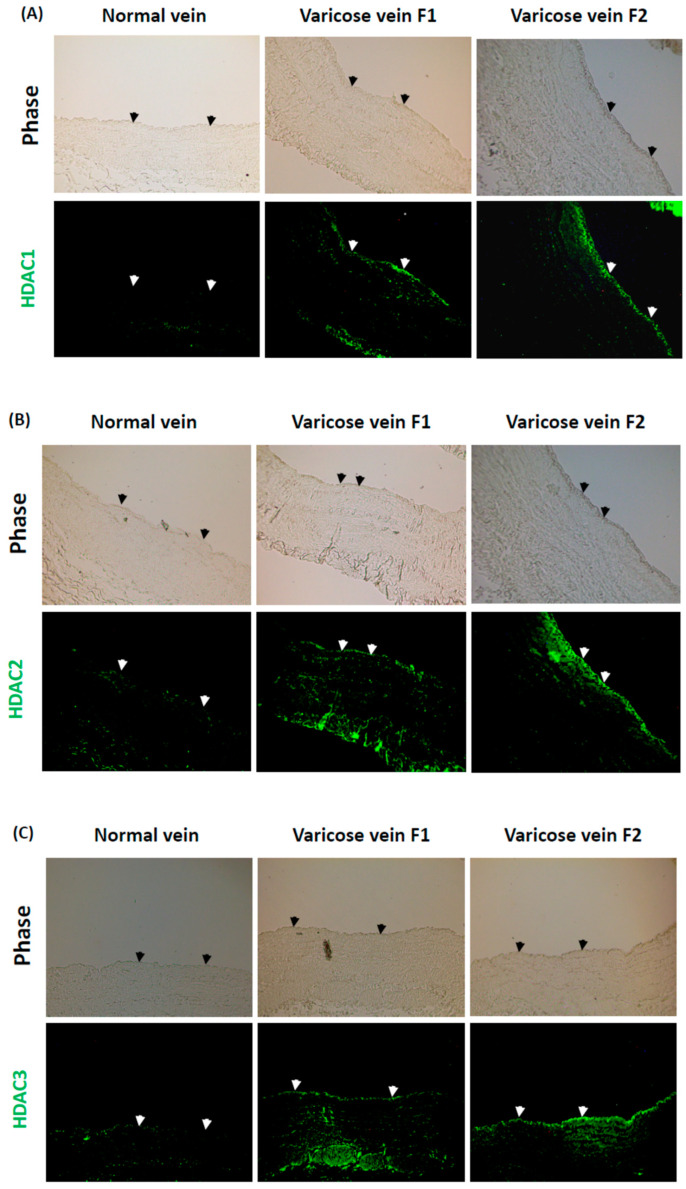
Class I histone deacetylases (HDACs) HDAC-1, -2, and -3 are predominantly expressed in the endothelium of human varicose veins with blood reflux but are rarely expressed in normal veins with normal venous flow. Cross-sections of human varicose veins with different levels of blood reflux (varicose vein F1 or F2) and human normal veins with normal venous flow (normal vein) were examined using immunostaining to detect the expression of human HDAC-1 (**A**), HDAC-2 (**B**), and HDAC-3 (**C**). Arrows represent the endothelia in the blood vessels. All photographs were obtained with fluorescence microscope with a 10× objective.

We previously demonstrated that Class I HDACs modulate quinone metabolism and vitamin A signaling to promote oxidation and inflammation in aortic ECs [19,20]. In this study, we investigated the expression of Class I HDACs in venous ECs in response to blood reflux in human CVD. The in vivo expression of HDAC-1, -2, and -3 in human varicose veins with different blood reflux levels was detected using immunostaining. The expression levels of these HDACs in normal human veins with normal venous flow were used as controls for comparison. The results showed that HDAC-1, -2, and -3 were highly expressed in the endothelium of human varicose veins with blood reflux (varicose vein F1). Moreover, the expression levels of HDAC-1, -2, and -3 were higher in human varicose veins with a longer duration time of blood reflux (varicose vein F2) (Figure 1A–C). In contrast, the expression levels of HDAC-1, -2, and -3 were low in the endothelium of normal veins (Figure 1A–C). These results suggest that Class I HDACs are highly expressed in venous ECs in response to blood reflux in human varicose veins but are rarely expressed in response to normal venous flow in normal human veins.

### 2.2. Class II HDACs in Venous ECs Are Upregulated in Response to Blood Reflux in Human Varicose Veins, but Downregulated in Response to Normal Venous Flow in Human Normal Veins

**Figure 2:**
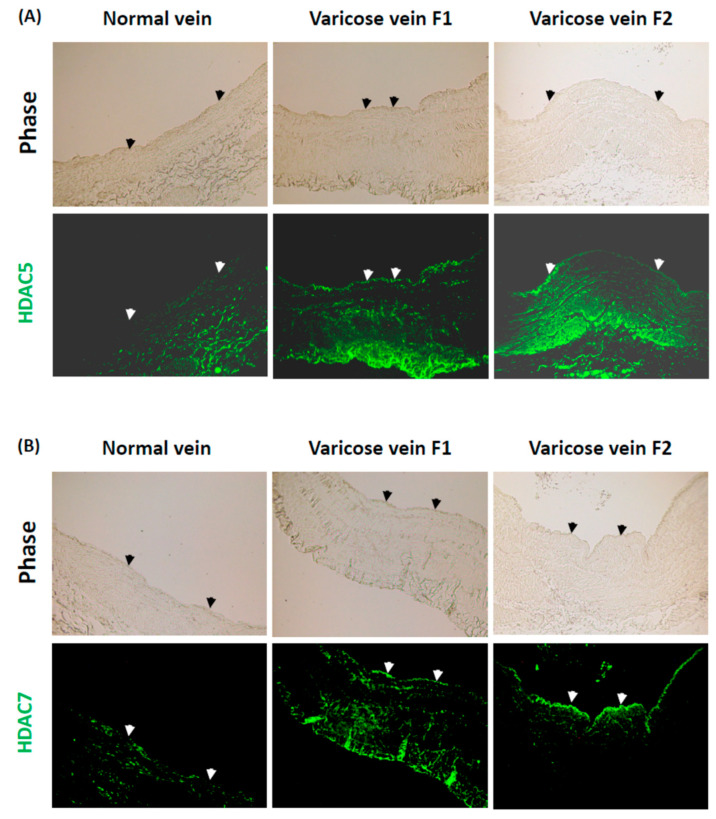
Class II HDACs (HDAC-5 and -7) are up-regulated in the endothelium of human varicose veins with blood reflux but are down-regulated in human normal veins with normal venous flow. Cross-sections of human varicose veins with different levels of blood reflux (varicose vein F1 and F2) and human normal veins with normal venous flow (normal vein) were immunostained for HDAC-5 (**A**) and HDAC-7 (**B**). Arrows represent the endothelia in the blood vessels. All photographs were obtained with fluorescence microscope with a 10× objective.

Class II HDACs inhibit vitamin A signaling and NOS expression to promote inflammation and NO repression in aortic ECs [19,20,21]. To identify the effects of blood reflux on Class II HDACs in CVD, we used immunostaining to compare the expression levels of Class II HDACs in human varicose veins with blood reflux and in human normal veins with normal venous flow. The results showed that HDAC-5 and -7 were upregulated in the endothelium of human varicose veins with blood reflux (varicose vein F1). The levels of blood reflux-induced HDAC-5 and -7 were enhanced in human varicose veins with a longer duration time of blood reflux (varicose vein F2) (Figure 2A,B). However, HDAC-5 and -7 were down-regulated in the endothelium of normal veins (Figure 2A,B). These results indicate that Class II HDACs are also upregulated in the endothelium of human varicose veins with blood reflux but are downregulated in human normal veins with normal venous flow.

### 2.3. Expression Levels of DNMTs in Venous ECs Are Overexpressed in Response to Blood Reflux in Varicose Veins, but Inhibited in Response to Normal Venous Flow in Normal Veins

**Figure 3:**
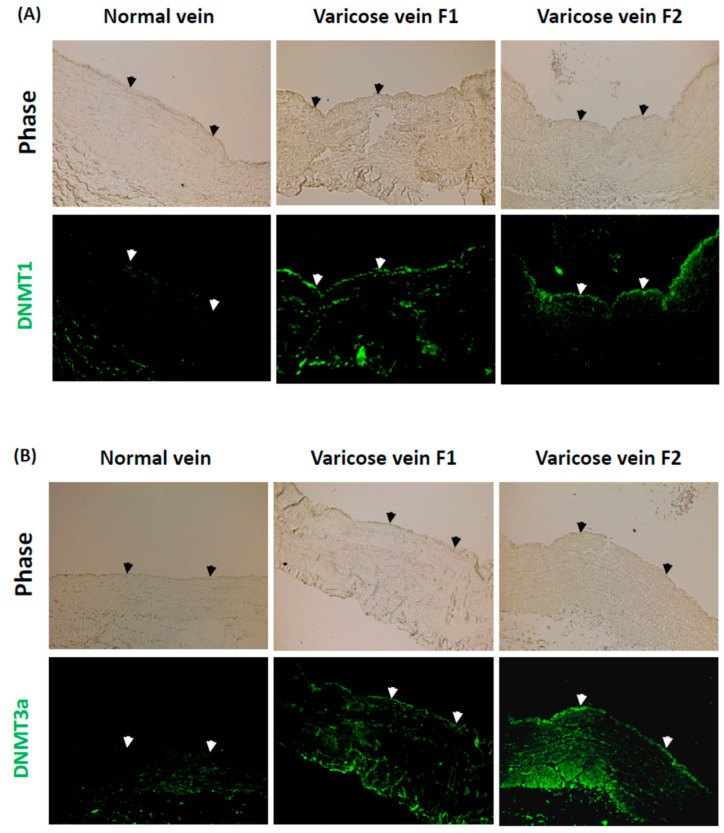
DNA methyltransferases (DNMTs; DNMT1 and DNMT3a) are over-expressed in the endothelium of human varicose veins with blood reflux but are inhibited in human normal veins with normal venous flow. Cross-sections of human varicose veins with different levels of blood reflux (varicose veins F1 and F2) and human normal veins with normal venous flow (normal vein) were immunostained for detecting DNMT1 (**A**) and DNMT3a (**B**). Arrows represent the endothelia in the blood vessel. All photographs were obtained with fluorescence microscope with a 10× objective.

DNMTs are vital epigenetic factors that can modulate NO production, proliferation, and inflammation in aortic ECs [22,23,24]. We aimed to elucidate the role of DNMTs in human varicose veins with blood reflux. The expression levels of DNMT1 and DNMT3a in human varicose veins with blood reflux were compared with those in normal human veins with normal venous flow. DNMT1 and DNMT3a were overexpressed in the endothelium of human varicose veins with blood reflux (varicose vein F1). The overexpression was greater in the varicose veins with a longer duration time of blood reflux (varicose vein F2) (Figure 3A,B). In contrast, the expression levels of DNMT1 and DNMT3a were inhibited in the endothelium of the normal vein (Figure 3A,B). These results indicate that DNMT1 and DNMT3a are over-expressed in the endothelium of human varicose veins with blood reflux but are inhibited in the endothelium of human normal veins with normal venous flow.

## 3. Discussion

We show that HDACs and DNMTs are overexpressed in venous ECs in response to blood reflux in vivo and are highly associated with the pathogenesis of CVD. Several lines of evidence support this conclusion. Immunostaining results demonstrate that Class I HDACs are predominantly expressed in the endothelial layer of human varicose veins with blood reflux in vivo but are rarely expressed in human normal veins with normal venous flow. Moreover, Class II HDACs are also upregulated in the endothelial layer of human varicose veins with blood reflux in vivo but are downregulated in venous ECs in response to normal venous flow in human normal veins. We further show that DNMTs are induced in venous ECs in response to blood reflux in human varicose veins but are inhibited in response to normal venous flow in human normal veins. Notably, blood reflux-induced HDACs and DNMTs in venous ECs were enhanced in human varicose veins with an increase in duration time of blood reflux (Summary in Table 1). Therefore, our findings provide a novel insight that HDACs and DNMTs are induced by blood reflux in venous ECs but are inhibited by normal venous flow. Blood reflux-induced HDACs and DNMTs are thought to modulate metabolic regulation to induce inflammation, NO repression, oxidation, and the proliferation of venous ECs to promote human veins toward CVD.

Veins have valves that enable only the forward travel of blood, generating normal venous flow and preventing blood reflux. The normal venous flow pattern, defined as laminar flow, can turn on EC protective signals to protect veins from diseases [2,3,25]. However, valve dysfunction creates blood reflux in CVD [2]. The flow pattern of blood reflux is identified as oscillatory flow, which can induce venous EC dysfunction, an inflammatory phenotype [26]. Clinically, blood reflux is defined as a vital pathophysiological finding for human CVD [27]. Moreover, a longer duration time of blood reflux is associated with more severe CVD [28,29]. This suggests that blood reflux-induced oscillatory flow develops in human CVD to promote venous EC dysfunction, whereas normal venous flow-induced laminar flow occurs in normal veins to maintain venous EC functions. Recently, metabolic regulation (vitamin regulation) has emerged as another vital factor affecting the development of CVD. The deficiency of vitamin A, C, or D has been reported to be correlated with the development of CVD in human patients [4,5,30,31]. In addition, vitamin C, D, or E supplementation has been shown to improve CVD [6,7,8,9]. Vitamins A, C, D, and E need to bind to their receptors, that is, retinoic acid receptors (RARs), vitamin D receptors (VDRs), vitamin C transporters (SVCT2), and PXR, respectively, to activate vitamin signaling and protect the functions of vascular cells [10,11,12]. This indicates that both blood reflux and metabolic regulation (vitamin signaling) affect CVD development. Thus, in this study, we used human varicose veins with different levels of blood reflux and normal veins with normal venous flow to mimic pathogenic and normal conditions, respectively, to identify the pathogenic mechanism of human CVD and correlate it with metabolic regulation.

HDACs are vital epigenetic factors; they repress metabolic regulators to modulate metabolic signaling and various cellular functions [13,14,15,16,17,19,20,21]. Class I HDACs (HDAC-1, -2, and -3) are one of the major HDAC groups involved in metabolic regulation and vascular functions [13,14,15,16,17,19,20,21]. We showed that HADC-3 is induced by proatherogenic oscillatory flow to associate with Class II HDACs (HDAC-5 and -7) to form a repressive complex with the vitamin A receptor RARα to inhibit vitamin A signaling and turn on inflammatory signaling in aortic ECs [19]. Class I HDACs are also induced by proatherogenic oscillatory flow to form an inhibitory complex that deacetylates the transcription factor Nrf2 to repress NQO1 expression, which can affect quinone metabolism and drive oxidative activity in aortic ECs [20]. In contrast, antiatherogenic laminar flow can acetylate Nrf2 to enhance NQO1 expression to regulate quinone metabolism and repress oxidative activity. In addition, the inhibition of Class I HDACs by HDAC inhibitors abolishes proatherogenic oscillatory flow-induced aortic EC proliferation in vivo [20]. These findings indicate that Class I HDACs can be modulated by hemodynamics to affect vitamin A signaling and quinone metabolism to promote the inflammation and oxidation of aortic ECs, resulting in aortic disease (atherosclerosis). Our present findings demonstrate that Class I HDACs are highly expressed in the endothelium of human varicose veins with blood reflux. Moreover, the expression levels of Class I HDACs were enhanced in human varicose veins with a longer duration time of blood reflux. In contrast, Class I HDACs were rarely expressed in the endothelium of normal human veins with normal venous flow. The flow pattern of blood reflux in veins is similar to the proatherogenic flow in aortic vessels (oscillatory flow), whereas the flow pattern of normal venous flow is similar to that of the antiatherogenic flow in aortic vessels (laminar flow) [25]. Compared with normal venous flow-induced laminar flow, blood reflux-induced oscillatory flow may have similar regulatory effects on proatherogenic oscillatory flow and antiatherogenic laminar flow in regulating the dysfunction or function of venous ECs. Taken together, these results suggest that Class I HDACs can be induced by blood reflux-induced oscillatory flow in venous ECs. Blood reflux-induced HDAC-1, -2, and -3 are predicted to modulate vitamin A signaling and quinone metabolism to turn on inflammation and oxidation and promote human CVD progression. In contrast, Class I HDAC-directed responses can be inhibited by normal venous flow-induced laminar flow to abolish inflammation and oxidation and protect human veins from CVD.

Class II HDACs are another major group of HDACs involved in metabolic regulation and vascular functions [13,14,15,16,17,19,20,21]. HDAC-5 and HDAC-7 are modulated by hemodynamics to affect vitamin A signaling and NO production, and thereby, affect metabolic regulation and cell functions in aortic ECs [19,20,21]. As previously mentioned, HDAC-5 and HDAC-7 were induced by proatherogenic oscillatory flow to associate with HDAC3 to form an inhibitory heterocomplex that inhibits the vitamin A receptor resulting in the repression of vitamin A signaling and activation of the inflammatory response in aortic ECs [19]. In contrast, antiatherogenic laminar flow can repress HDAC-5 and -7 to form an inhibitory complex to activate vitamin A protective signaling [19]. As demonstrated by Wang et al. and our group, proatherogenic flow and antiatherogenic laminar flow have differential effects on the association of HDAC-3/-5/-7 and the transcription factor MEF2 to modulate the expression of KLF2, a vital transcriptional factor that regulates the metabolic state and induces NO production to promote normal functions in aortic ECs [18,19,20,21]. In this study, we found that Class II HDACs were upregulated in the endothelium of human varicose veins with blood reflux. Blood reflux-induced Class II HDACs in venous ECs were enhanced in the human varicose veins with a longer duration time of blood reflux. However, Class II HDACs were downregulated in normal human veins with normal venous flow. These findings suggest that Class II HDACs can be induced by blood reflux-induced oscillatory flow to affect NOS and vitamin A signaling, leading to NO repression and inflammation in venous ECs that promotes the development of CVD. In contrast, Class II HDACs can be inhibited by normal venous flow-induced laminar flow to abolish these responses and prevent CVD development.

DNMTs are the other important epigenetic factors that affect metabolic regulation and various vascular functions [14,22,23,24,32]. DNMT1 and DNMT3a are two major DNMTs in ECs that direct the methylation of genes or signaling molecules to modulate aortic disorders, such as atherosclerosis [14]. In vivo and in vitro studies have shown that a high-fat diet or high LDL cholesterol can induce DNMT1 expression [33]. More specifically, DNMT1 and DNMT3a are involved in hemodynamics-modulated metabolic regulations in aortic ECs [14,22,23,24]. DNMT3a was found to be induced by proatherogenic oscillatory flow to direct methylation in the KLF4 promoter and repress its expression and subsequently inhibit NOS3 expression and NO production in aortic ECs in vitro. These effects of DNMT3a were also confirmed in vivo [22]. In addition, DNMT1 is induced by proatherogenic oscillatory flow to enhance the expression of cyclin A and connective tissue growth factor to promote proliferation and inflammation, respectively, in aortic ECs [23,24]. The abolishment of DNMT1 in vivo can inhibit the formation of aortic disease (atherosclerosis) [24]. We found that both DNMT1 and DNMT3a were overexpressed in the endothelium of human varicose veins with blood reflux. The overexpression of DNMT1 and DNMT3a was enhanced in the varicose veins with a longer duration time of blood reflux. In contrast, the DNMT1 and DNMT3a expression levels were inhibited in normal human veins with normal venous flow. These results suggest that DNMT1 and DNMT3a were induced by blood reflux-induced oscillatory flow to promote NO repression, inflammation, and proliferation in venous ECs, which can promote the development of human CVD. However, the expression of DNMT1 and DNMT3a was abolished by normal venous flow-induced laminar flow to inhibit these responses in venous ECs and protect human veins from CVD.

## 4. Materials and Methods

### 4.1. Human Veins

Diseased human varicose veins were collected from patients with varicose veins at the National Taiwan University Hsin-Chu Hospital in Taiwan. Varicose veins were in the early stage (C2–C3 degree of CEAP classification) of CVD. The compression–release method and ultrasound were used to detect whether blood reflux was generated. The level of blood reflux was evaluated based on the duration time of reflux. The veins exhibited different levels of blood reflux where a duration time of 1 s indicated an varicose vein F1 and more than 2 s for varicose vein F2. Normal human saphenous veins were collected from bypass patients as normal veins with normal venous flow (normal vein). The compression-release method and ultrasound were used to confirm that blood reflux was not generated in the normal vein.

### 4.2. Detection of Class I HDACs (HDAC-1, -2, and -3), Class II HDACs (HDAC-5 and -7), and DNMTs (DNMT1 and DNMT3a) in Human Varicose Veins with Blood Reflux in Comparison with Human Normal Veins with Normal Venous Flow

Human varicose veins with different levels of blood reflux (varicose veins F1 and F2) and normal veins with normal venous flow (normal vein) were collected. They were fixed in formalin and embedded in paraffin blocks. The expression of Class I HDACs (HDAC-1, -2, and -3), Class II HDACs (HDAC-5 and -7), and DNMTs (DNMT1 and DNMT3a) in these samples was examined using immunostaining.

### 4.3. Immunostaining

Immunostaining was performed as previously described [34]. The cross-sections of varicose vein F1 and F2, and normal veins were examined using immunostaining for detecting the expression of Class I HDACs (HDAC-1, -2, and -3), Class II HDACs (HDAC-5 and -7), and DNMTs (i.e., DNMT1 and DNMT3a). Briefly, sections of these veins were deparaffinized and blocked for 1 h with serum albumin dissolved in phosphate-buffered saline (5 mg/mL). The sections were incubated with either an anti-HDAC-1 antibody (Abcam, Waltham, MA, USA), anti-HDAC-2 antibody (Abcam, Waltham, MA, USA), anti-HDAC-3 antibody (Abcam, Waltham, MA, USA), anti-HDAC-5 antibody (Abcam, Waltham, MA, USA), anti-HDAC-7 antibody (Abcam, Waltham, MA, USA), anti-DNMT1 antibody (Santa Cruz, CA, USA), or anti-DNMT3a antibody (Santa Cruz, CA, USA) (1:50) for 1 h at 37 °C and subsequently with FITC-conjugated secondary antibody (1:1000) for 2 h at room temperature. The slides were mounted and the images were captured using a fluorescence microscope.

## 5. Conclusions

We have elucidated the relationship between the epigenetic factors HDACs -1, -2, -3, -5, and -7, as well as DNMT1 and DNMT3a, and blood reflux (the vital pathogenic factor for CVD) in human CVD. We compared the in vivo expression of HDACs and DNMTs in human varicose veins with blood reflux with that in human normal veins with normal venous flow to demonstrate that both HDACs and DNMTs are induced in human venous ECs in response to blood reflux. The expression of blood reflux-induced HDACs and DNMTs is enhanced in human varicose veins with a longer duration time of blood reflux. HDACs and DNMTs are anticipated to modulate metabolic regulation (vitamin A signaling, NOS expression, and quinone metabolism) to turn on inflammation, NO repression, oxidation, and proliferation in venous ECs, which are highly associated with the promotion in the progression of human CVD. In contrast, HDACs and DNMTs are inhibited in human venous ECs in response to normal venous flow to prevent human veins from CVD. These results show the potential role of HDACs and DNMTs in the metabolic regulation of CVD pathogenesis. This study provides new knowledge that blood reflux-induced epigenetic factors HDACs and DNMTs are involved in the pathogenesis of venous EC dysfunction in human CVD. These findings will facilitate the diagnosis and therapy of human CVD. Detection of HDACs and DNMTs in venous ECs has the potential to be developed as a new diagnostic biomarker for human CVD. Moreover, blood reflux-induced HDACs and DNMTs can be potential targets for the therapy of human CVD, and their inhibitors could have therapeutic potential for CVD.

## Figures and Tables

**Table 1:** Comparison of the expression of epigenetic factors HDACs and DNMTs in venous endothelial cells of normal veins with normal venous flow and in the endothelial cells of varicose veins with different levels of blood reflux.

	Normal Vein	Varicose Vein F1	Varicose Vein F2
HDAC-1	-	+	+++
HDAC-2	-	+	+++
HDAC-3	-	+	+++
HDAC-5	-	+	+++
HDAC-7	-	+	+++
DNMT1	-	+	+++
DNMT3a	-	+	+++

+: The expression level of epigenetic factor HDAC or DNMT. +++: The expression level of epigenetic factor HDAC or DNMT was more dominant.

## Data Availability

The data used to support the findings of this study are included within the article.
